# Taking the Occam’s Razor Approach to Hedgehog Lipidation and Its Role in Development

**DOI:** 10.3390/jdb6010003

**Published:** 2018-01-30

**Authors:** Dominique Manikowski, Philipp Kastl, Kay Grobe

**Affiliations:** Institute of Physiological Chemistry and Pathobiochemistry and Cells-in-Motion Cluster of Excellence, University of Münster, D-48149 Münster, Germany; d.manikowski@uni-muenster.de (D.M.); p_schu24@uni-muenster.de (P.K.)

**Keywords:** Hedgehog, morphogen transport, posttranslational modification

## Abstract

All Hedgehog (Hh) proteins signal from producing cells to distant receiving cells despite being synthesized as N-and C-terminally lipidated, membrane-tethered molecules. To explain this paradoxical situation, over the past 15 years, several hypotheses have been postulated that tie directly into this property, such as Hh transport on cellular extensions called cytonemes or on secreted vesicles called lipophorins and exosomes. The alternative situation that tight membrane association merely serves to prevent unregulated Hh solubilization has been addressed by biochemical and structural studies suggesting Hh extraction from the membrane or proteolytic Hh release. While some of these models may act in different organisms, tissues or developmental programs, others may act together to specify Hh short- and long-range signaling in the same tissues. To test and rank these possibilities, we here review major models of Hh release and transport and hypothesize that the (bio)chemical and physical properties of firmly established, homologous, and functionally essential biochemical Hh modifications are adapted to specify and determine interdependent steps of Hh release, transport and signaling, while ruling out other steps. This is also described by the term “congruence”, meaning that the logical combination of biochemical Hh modifications can reveal their true functional implications. This combined approach reveals potential links between models of Hh release and transport that were previously regarded as unrelated, thereby expanding our view of how Hhs can steer development in a simple, yet extremely versatile, manner.

## 1. Hedgehog (Hh) Proteins: Extensively Post-Translationally Modified Signals

Cellular communication is essential in multicellular organisms to coordinate both development and homeostasis in adult life. To this end, the signalling cell often releases soluble molecules that travel to and bind to receptors expressed by the target cell. Subsequent intracellular signal transmission requires sequential interactions between ligand-activated cell-surface receptors, adaptors, proteins of the signal transduction cascade and transcription factors. Most of these intracellular proteins are switched on or off by post-translational protein modifications (PTMs), such as phosphorylation, lipidation and proteolytic processing or degradation, but also by association with other proteins and thereby compartmentalization within cells. Events in the extracellular matrix (ECM) that regulate the release and transport of signalling molecules have also become recognized as important regulators of cellular communication. The Hedgehog (Hh) family is one important example of extracellular signalling molecules that undergo several PTMs during biosynthesis [1]. The most important Hh PTM is conserved dual lipidation, which firmly tethers Hh molecules to the outer membrane leaflet of the cell that produces them. Lipidation is also essential for Hh signalling to its receptor Patched (Ptc) on target cells over both short (2–3 cell diameters in the *Drosophila melanogaster* wing disc) and long distances (up to 12–15 cell diameters in wing discs and vertebrate tissues such as the developing neural tube and developing limbs) [2,3,4,5]. Over the past two decades, genetic, biochemical and advanced imaging techniques have been extensively used to resolve the paradoxical situation that an insoluble lipidated molecule can travel to target cells at significant distances from its source, where post-translational lipidation normally restricts protein movement. In this review, we use the problem-solving principle of parsimony, or Occam’s razor, to discuss these obtained major models of Hh release and transport. Occam’s razor originally stated: “Plurality should not be posited without necessity”, i.e., exclude concepts, variables or constructs that are not needed to explain a phenomenon. An important condition in the Occam’s razor concept is that the remaining explanations must strictly accommodate all available evidence: An explanation is to be dismissed if it fails to account for the established evidence and, among those explanations that are in agreement with the evidence, Occam’s razor favours the “simpler” one, i.e., the explanation requiring the fewest leaps of logic. Although we find that no single model fulfils these criteria completely, their combination can. This provides new hypotheses for future exploration.

## 2. Conserved Post-Translational Hh Lipidation

Hhs were discovered more than 35 years ago in a screen for genes involved in the developmental segmentation of *Drosophila* embryos [6]. In vertebrates, three orthologues were identified: Sonic hedgehog (Shh), Indian hedgehog (Ihh) and Desert hedgehog (Dhh)*.* These proteins differ primarily in their tissue distribution. Shh is expressed in epithelia and in the developing nervous system [7,8] as well as in the thymic stroma and foetal liver to regulate lymphocyte development [9,10,11] (summarized in [12]). Ihh is expressed in the developing bone [13] and during T-cell development in foetal and adult thymus [14] as well as during T-cell receptor activation of naïve CD8 cells and cytotoxic T lymphocytes [15]. Dhh is expressed in the mouse spleen to regulate multiple stages of erythrocyte differentiation [16] and in the peripheral nervous system and reproductive organs [17] to regulate their function by localized signalling [18]. Because of its widespread distribution and expression in three key signalling centres in vertebrate embryos (the notochord, the floor plate and the zone of polarizing activity), Shh is the most extensively studied vertebrate Hh family member. Yet, all Hhs activate the same conserved signalling pathway downstream of Ptc receptors to regulate embryonic patterning in vertebrate and invertebrate embryos [19,20], as well as stem and progenitor cell populations in the adult [21,22]. Shh misexpression leads to several forms of cancer [23], and loss of Shh function causes developmental midline defects in mice, chicks and humans [24,25,26,27]. Notably, multiple Hh PTMs contribute significantly to these Hh functions. 

**Figure 1:**
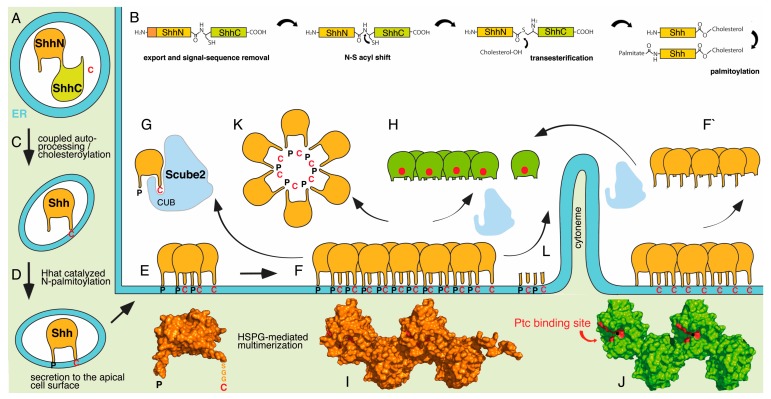
Biosynthesis and release models of dual-lipidated Hh, using vertebrate Shh as an example. The 45 kDa ShhNC precursors are secreted into the ER and their signal sequence is processed (**A**). This is followed by cholesterol esterification of the C-terminal peptide by intein-related autoprocessing/cholesterol esterification (**B**,**C**). Subsequent palmitoylation of N-terminal peptides is catalysed by Hhat. This generates bioactive dual-lipidated Shh (**D**). After export to the cell surface, Shh firmly tethers to the outer membrane leaflet of the plasma membrane (**E**) and generates large clusters (**F**), using cell-surface-associated HSPGs as scaffolds (not shown for clarity). Crystal lattice interactions, biochemical assays and in vitro cell culture assays suggested that N-terminal peptides of one molecule in the cluster block Ptc receptor binding sites (red) of its neighbour, rendering the molecule inactive. Several Shh release mechanisms have been proposed: direct, Scube2-mediated cholesterol extraction from the membrane (**G**), proteolytic processing of both lipidated peptides (**H**), micelle formation (**K**) or Hh piggybacking on exosomes or lipoprotein particles and transport on extended, filopodia-like structures called cytonemes (**L**). Note that one way to explain strongly reduced bioactivity of non-palmitoylated Shh is that proteolytic processing of Shh N-termini is impaired during release, resulting in soluble clusters with their receptor binding sites still blocked (**F’**,**I**). Proteolytic processing, in contrast, reverses the Ptc blockade by N-terminal peptides in the cluster (**J**) and thereby activates the protein. The Scube2-assisted in vitro conversion of inactive unprocessed Shh into truncated fully bioactive Shh supported this mechanism (**F**–**H**). I, J: Tetrameric Shh crystal lattice interactions as part of a larger extended structure are shown. P: palmitate, C: cholesterol.

Hh PTMs start in the endoplasmic reticulum (ER) with the removal of the signal sequence. The resulting ~45 kDa pro-proteins consist of an N-terminal signalling domain that starts with a cysteine (C24 in human nomenclature, C25 in mouse, C85 in *Drosophila*) and a C-terminal autoprocessing/cholesterol transferase domain (called HhC/ShhC [28]) (Figure 1A). In a reaction analogous to intein-mediated protein splicing, ShhC amino acid C198 (human nomenclature, corresponding to C258 in *Drosophila* HhC) undergoes an acyl rearrangement (replacement of a peptide bond with a thioester bond) followed by a transesterification reaction (attack of the thioester bond by the hydroxyl group of a cholesterol moiety) (Figure 1B). This unique reaction covalently couples cholesterol to the C-terminus of the N-terminal signalling domain and simultaneously splits the ~45-kDa pro-protein at the cholesteroylation site [29] (Figure 1B,C). The sole known role of HhC is to catalyse this reaction, consistent with its high sequence similarity to inteins [30]. Therefore, HhC has redirected the ability of inteins to ligate flanking peptides to the quantitative covalent ligation of HhN peptides with cholesterol [31].

As a consequence of mechanistically coupled Hh cholesteroylation/autoprocessing, Hhs are the only known proteins secreted as C-cholesteroylated 19 kDa proteins, and mutations that prevent this reaction result in severe developmental defects [26,32]. Unlike the C-terminal autoprocessing domain that is rapidly removed by ER-associated degradation, the cholesteroylated signalling domain undergoes a second post-translational lipidation, called N-palmitoylation, at the amino terminus of the most N-terminal conserved cysteine [33] (Figure 1B,D). This irreversible attachment of the 16-carbon fatty acid palmitate requires the enzymatic activity of a separate enzyme, called Hedgehog acyltransferase (Hhat in mammals or Ski in *Drosophila melanogaster*) [34,35]. The resulting dual-lipidated Hh protein is secreted onto the cell surface (Figure 1E) and locates to sterol-rich membrane microdomains, also called lipid rafts [36]. Although C-terminal cholesteroylation [37] and palmitoylation [38,39] are expected to tightly anchor the protein to cell membranes, dual-lipidated Hh is highly bioactive at both short and long ranges, and both lipids affect Hh transport and signalling activity but in different manners. Engineered HhN that lacks cholesterol is bioactive and is efficiently secreted from the producing cell in vitro [40,41,42,43], but when expressed in vivo, it fails to concentrate near the HhN-secreting cells and travels too far from the source of secretion, resulting in aberrant tissue patterning [32,41,44,45]. This suggests that rather than regulating the affinity of Hh proteins for their Ptc receptors, cholesterol restricts Hh movement through tissues [32]. By contrast, N-palmitoylation regulates Hh bioactivity [33,43,46,47,48]. In cultured cells that express the Hh receptor Ptc, the dual-lipidated protein is 30–800 times as active as non-palmitoylated Shh [33,49], and non-palmitoylated *Drosophila* Hh is completely inactive in vivo [42,50]. In vivo, N-palmitate in vertebrates seems to be less important: Ectopic overexpression of non-palmitoylated Shh in the mouse induces significant gain-of-function phenotypes [46,47,50], and loss of palmitoylase activity in Hhat mutants causes defects that are characteristic of defective Shh signalling, yet are less severe than Shh loss-of-function phenotypes [46]. Several models have been proposed to explain the various effects of dual Hh lipidation on its function. One broadly accepted idea is that Hh lipidation is essential for membrane association, which is a prerequisite for subsequent Hh interaction with factors required for its regulated transport.

## 3. Hh Lipidation as a Prerequisite for Cell-Autonomous Hh Interactions at the Cell Surface

One firmly established accessory protein that interacts with lipidated Hh at its site of secretion is the transmembrane protein Dispatched (Disp) [51]. Mice and flies deficient in Disp synthesize Shh/Hh but fail to release the protein from the surface of producing cells, resulting in developmental phenotypes similar to those seen in Shh/Hh knockouts [51,52]. Consistent with Disp containing a sterol-sensing domain, it releases cholesterol-modified Shh but is not required for the secretion of genetically engineered ShhN variants that lack C-cholesterol [51]. This indicates that in addition to firmly tethering Hh to the membrane of the cell, C-cholesterol may be involved in Hh solubilization by Disp. To date, however, the subcellular localization and exact mode of Disp function is not clear, in part because Disp alone is insufficient to solubilize Shh [53]. This finding suggests that cofactors are required to form a functional Hh release complex at the cell surface.

One such group of cofactors are the cell-surface heparan sulfate (HS) proteoglycans (HSPGs) of the glypican family. Glypican HSPGs are composed of a glycosylphosphatidyl-inositol (GPI)-linked protein core to which varying numbers of linear glycosaminoglycan chains are covalently attached. Mutations in genes required for the biosynthesis of cell-surface-associated glypican core proteins and HS chains severely attenuate Hh signalling in vivo [54,55,56]. Two established HSPG functions may explain attenuated Hh signalling: First, HSPGs act as scaffolds for the assembly of large, light microscopically visible Hh clusters at the cell surface [57,58] (Figure 1F) that are up to 50-fold more potent than monomeric HhN in activating biological responses [46]. Second, cell clones deficient in the HSPG biosynthetic or modifying genes tout-velu (required for biosynthesis of the HS glycosaminoglycan backbone) and sulfateless (required for HS sulfation) completely block Hh transport to receiving cells in Drosophila wing discs [54], and HSPG-modifying sulfatase enzymes (that selectively desulfate HS) also modulate Hh transport [59,60]. These observations indicate that HSPGs are essential direct or indirect mediators of Hh multimerization and transport that act up to 50 µm from the Hh source in Drosophila imaginal wing discs and up to 300 µm from the source in vertebrate limbs [5]. 

## 4. Hh Lipidation as a Prerequisite for Cell-Non-Autonomous Hh Interactions with Soluble Molecules

In addition to cell-autonomous Disp and HSPG functions, several cell-non-autonomous mechanisms help release Shh from the surface of vertebrate cells. One example of Shh release factors that act in trans are the secreted Scube (Signal peptide, CUB-EGF-like domain containing protein) glycoprotein homologues 1–3. Scube1 is highly expressed in liver, lung and brain [61], while Scube2 is more broadly expressed [62], and Scube3 is expressed in bones and osteoblasts [63]. Despite the differential expression of Scube1–3, their structural topology is highly conserved, suggesting a similar function. All Scube homologues consist of nine N-terminal epidermal growth factor (EGF)-like domains, followed by a spacer region, three cysteine-rich domains and a C-terminal CUB domain [64,65,66]. In vivo, Scube functions were revealed in a functional screen for Hh-related (you-class) phenotypes in the zebra fish, “sonic you” representing Shh in this species [66,67]. Morpholino (MO)-mediated knockdown of Scube2 activity in the zebra fish embryo leads to mild defects in Hh signalling [64], and the simultaneous knockdown of all three Scube homologues completely abrogates Hh signalling [68]. Finally, recombinant Scube2 was found to increase Shh release from the surface of producing cells by about ten-fold [64,65,66,69], an activity for which the CUB domain is required in vitro [53,70,71] and in vivo [72]. Another region essential for Scube-mediated Shh release is the spacer region [62], which specifically binds physiological HS and thereby co-localizes the Scube2 release factor with the HSPG-associated Shh substrate at the cell surface [73,74].

## 5. Proposed Model of Direct Disp- and Scube2-Mediated Shh Extraction from the Producing Cell

How can Scube2 CUB domain-dependent Shh solubilisation from the cell surface be mechanistically explained? One idea is that the release of dual-lipidated, and thus hydrophobic, Shh into the hydrophilic extracellular environment requires Shh extraction from the membrane and subsequent lipid sequestration to permit transport. One recent model proposed transfer of membrane-associated Shh to soluble Scube2 via Disp and suggested that cholesterol modification is sufficient to drive this interaction [53]. Scube2 then continues to “chaperone” the extracted cholesterol away from the aqueous environment to transport Shh to the receiving cell [53] (Figure 1G). Another study showed that Scube2 solubilizes dual-lipidated Shh from cultured cells or purified detergent-resistant membrane microdomains and that Shh solubilisation is enhanced by the palmitate adduct and by Disp [70]. Both studies confirm the essential role of the Scube2 CUB domain in Shh release, possibly by cholesterol sequestration [53], which suggests a mechanism for lipid-modified Shh signalling at distal sites, in agreement with established protein transport modes [39]. The proposed model explains the essential signalling function of N-palmitate by its continued exposure during Scube2/Shh transport to allow for its subsequent direct interaction with the Hh receptor Ptc [75].

This model generates several predictions that can be assessed for their congruence with established properties of Hh PTMs. The first prediction is that Scube2-mediated Hh extraction can occur despite being highly endergonic. The removal of a single cholesterol from a lipid bilayer requires approximately 82 kJ/mol [76,77]. Hence, the extraction of cholesteroylated Hh hexamers would require 6 × 82 kJ/mol (approximately 500 kJ/mol), and approximately 1.6 MJ/mol would be required to extract average-sized 400-kDa Hh clusters from the cell surface [58]. These thermodynamic constraints, together with the lack of extracellular energy donors that could theoretically drive the process, make protein solubilisation through extraction unlikely. The other possibility, that Disp consumes intracellular ATP to extract and hand over Shh to Scube2, is ruled out by reported Disp-independent Shh release [53] and Scube2-enhanced Shh release in cell-free systems [70]. The second prediction is that the N-palmitate of membrane-associated Hh clusters [46,78,79] (Figure 1F) does not prevent Hh solubilisation, which, however, is not in line with its hydrophobicity and established membrane association [38,80]. The third prediction—that N-palmitate plays a direct role in receptor binding [75]—is at odds with largely unimpaired signalling of Hh variants that were N-terminally linked to a variety of long-chain fatty acids or hydrophobic amino acids [48,81], as these will not bind to Ptc. Finally, and most importantly, the lack of Scube orthologues in *Drosophila* suggests completely different mechanisms of Hh release in vertebrates and invertebrates, despite otherwise similar signalling modes. Therefore, the proposed model of direct Shh extraction makes a number of assumptions about the roles of Hh PTMs, especially its dual lipidations, and does not account for thermodynamic constraints of Hh multimer extraction from the membrane (Table 1).

## 6. Proposed Model of Indirect Scube2 Effects as Enhancers of Proteolytic Shh Processing from the Surface of Producing Cells

An alternative indirect mechanism of Scube2 CUB domain-dependent Shh release is suggested by the CUB acronym, which is derived from the complement serine proteinases C1r, C1s, MASP-1, MASP-2 and MASP-3; UEGF; and bone morphogenetic protein-1/tolloid metalloproteinases (consisting of the four members BMP-1, mTLD, mTLL-1 and mTLL-2) [82]. The essential role of CUB domains in these proteinases is to mediate specific protease/substrate complex assembly to promote regulated substrate proteolysis [83]. Other examples of CUB-regulated proteolytic activators are the procollagen C-proteinase enhancers (PCPEs), which stimulate C-terminal processing of fibrillar procollagens by CUB domain-mediated binding to the substrate [82,84]. However, PCPEs are devoid of intrinsic proteolytic activity, and instead function by changing the procollagen structure, which in turn results in a ten-fold increase in tolloid proteinase binding and cleavage of the pro-domain [85]. Notably, this activity is solely a property of the CUB domain [84,86]. Both PCPEs and Scube proteins strongly bind to HSPGs [73,74,87], and polysulphated glycans super-stimulate PCPE-regulated proteolytic procollagen processing [88] and Shh solubilisation [29,57]. Hence, it is possible that Scube2 CUB domains also act as protease activators for the regulated cleavage of both lipidated Shh termini from the globular Shh signalling domain (Figure 1H). This activity would release the morphogen from the cell surface as a prerequisite for its short- and long-range transport.

Indeed, both proteolytic Shh release from the surface of producing cells and Scube2-mediated enhanced Shh processing have been reported [71,89,90,91]. These reports generate several predictions that can be assessed for their congruence with established functions of Hh PTMs. The first prediction—that Hh lipids tether the unprocessed protein to the cell membrane to restrict its spread until Hh release is required—is fulfilled [33,92], and the role of Hh N-palmitate in the process is comparable to that of the *Drosophila* EGF receptor ligand, Spitz. Here, the same acyltransferase that palmitoylates Hh also palmitoylates N-terminal cysteine residue 29 of Spitz to promote its association with the extracellular leaflet of the plasma membrane [93,94]. Because N-palmitoylation is resistant to all known palmitoylate esterase activities [38,95], theoretical possibilities for solubilising Spitz are restricted to proteolytic processing of its palmitoylated N-peptide. Consistent with this, the sequence of wild-type soluble Spitz in vitro begins at methionine 45, whereas a palmitoylation-deficient mutant (Spitz^C29S^) remains unprocessed, starting with serine 29 [93], as a consequence of its direct secretion from the cell. Proteolytic Spitz release is also consistent with the unimpaired capacity of non-palmitoylated Spitz to bind to and activate its receptor in vitro (reviewed by [96]), and its release from the membrane is consistent with the reported solubilisation of the mammalian Spitz orthologue, transforming growth factor-α by the cell-surface-associated protease ADAM17 [97]. 

Therefore, the second prediction is that proteolytically processed Hhs are also bioactive and lack palmitoylated N-terminal peptides, as shown for Spitz. Indeed, N-terminally truncated soluble and bioactive vertebrate Shh and invertebrate Hh have been described [91,92,98]. This finding, however, seems to be at odds with the established essential role of N-palmitate for Hh biofunction, as demonstrated by genetic Hhat inactivation [78,79] and site-directed mutagenesis of the conserved palmitate acceptor cysteine [33,43]. A solution to this paradox came from the observation that unprocessed N-terminal peptides block Ptc receptor binding sites of adjacent molecules in the multimeric cluster (Figure 1F) and that their proteolytic removal exposes these sites to activate the truncated protein [90,91] (Figure 1H). Because of its continued membrane association, the essential yet indirect role of N-palmitate is to ensure that only fully processed Hh clusters are released from the cell surface and are simultaneously primed for Ptc binding at the surface of target cells. As a consequence of this mechanism, genetic or pharmaceutical inhibition of Hh palmitoylation results in the release of N-terminally unprocessed proteins (similar to Spitz^C29S^ secretion) with their Ptc binding sites still blocked [90] (Figure 1F’). In contrast, by using a bicistronic Shh/Hhat co-expression system to enhance otherwise low Shh N-palmitoylation to almost complete levels in vitro, soluble protein clusters lacked almost all N-terminal peptides and were highly bioactive [71]. Coupled N-terminal Hh processing and activation is supported by crystal lattice interactions observed in the protein database structure 3m1n [90,99] (Figure 1I). Here, unprocessed N-terminal extended peptides locate to the Hh binding site for its receptor Ptc and the Hh inhibitory 5E1 antibody that binds to the same site [100,101,102] (Figure 1J). Finally, and notably, Scube2-assisted removal of non-palmitoylated Shh^C25A^ peptides restored Shh biofunction in vitro [91]. This finding supports the suggestion that N-palmitate is not required for direct Ptc binding and Hh signalling at the receiving cell and explains how non-palmitoylated Shh^C25S^ can retain significant inductive and patterning activity in vertebrate tissues in vivo, as described previously [46,49,50,91] (Table 1).

The third prediction implied by proteolytic Hh cleavage—that thermodynamics restricts all reactions in the ECM to exergonic modes, such as peptide cleavage—is also fulfilled. Proteolytic Hh processing is thus in accordance with Occam’s razor in several ways: First, no unnecessary assumptions need to be made about the mechanisms of Hh release from the membrane, its energy requirements, and the role of CUB domains in the process, because Shh processing resembles that of other lipidated proteins, such as Spitz and Wnt [103]. Virtually all proteins can be shed if they have a globular domain at some distance from the cell membrane, and there is no reason to assume that Hh is an exception: Here, cholesterol tethers Hh to membranes [37] to prevent leakage and accidental perturbation of the Hh pathway by non-lipidated bioactive HhN [32], and N-palmitate controls Hh bioactivation. The congruence principle is also fulfilled because direct functional links exist between quantitative Hh cholesteroylation (to tether all 19-kDa Hh to the plasma membrane), HSPG-assisted Hh multimerization (which assembles and stores inactive Hh clusters and recruits Scubes for their release [57,73]), Scube2 CUB domain-regulated proteolytic processing [91] and irreversible Hh N-palmitoylation (making Hhs resistant to the alternative possibility of esterase-mediated release [95,98]). Therefore, Hh lipidation, HSPG functions and Scube2-assisted proteolytic processing can be combined without problems into one interdependent multi-step mechanism of Shh release and activation. In further support of this combined mode, Chamberlain et al. reported that MβCD, a ringed compound that extracts cholesterol from cell membranes, releases Shh and a green fluorescent protein (GFP)-tagged Shh variant into the media of expressing cells [104]. Although the authors suggested MβCD solubilisation of these proteins by direct extraction of the Shh cholesterol, this mechanism is not in line with the molecular mechanism of MβCD cholesterol extraction [76,77]. MβCD association with the membrane surface destabilizes the local packaging of cholesterol and favours cholesterol uptake into the MβCD dimer, but only if MβCD is positioned right above the cholesterol. Such MβCD positioning, however, is made impossible by the cholesterol-linked C-terminal Hh protein stalk that blocks the site above the cholesterol. Instead, MβCD depletes membranes of free cholesterol, perturbs lipid rafts, and thereby increases proteolytic protein processing at the membrane [105]. 

HSPG-modulated shedding also represents a useful model to explain functional links between cell-surface HS biosynthesis and Hh bioactivation in lung [106], spinal cord [60] and fly wing [59] development in vivo. Moreover, Scube2-regulated shedding provides an explanation for the compensated loss of embryonic Hh function as a consequence of simultaneous knockdown of all three Scube genes in triple MO embryos [68] by increased ligand expression. Shh mRNA injection into Scube triple MO embryos rescued you-class phenotypes, and a moderate (four-fold) Shh mRNA increase induced wild-type-like ectopic Shh target gene expression in these embryos [68]. These in vivo observations are incompatible with direct Scube-mediated Shh extraction and continued association during transport because any Scube1-3 extraction and transport blockade would not simply be bypassed by increased amounts of ligand. By contrast, baseline Shh processing in the absence of Scube is influenced by the frequency of random protease/substrate encounters that increase with increasing concentrations of Shh ligand and protease. Scube enhancer function also explains the tissue-restricted phenotypes in you-class zebrafish mutants [65] and in Scube-deficient mice [61,107], the lack of Scube orthologues in *Drosophila* [53] and the CUB domain-independent release of Shh mutants made prone to proteolytic processing [91]. Furthermore, the role of the N-terminal lipid—to anchor the unprocessed protein to the cell surface, as opposed to a direct role in receptor binding [75]—explains why it can be functionally replaced by a wide variety of hydrophobic residues ranging from long-chain fatty acids to hydrophobic amino acids [48,81]. An important limitation to the model of proteolytic Hh solubilisation, however, is that diffusive Hh transport is the only possibility to relay the released protein from producing to receiving cells in vivo. Yet, timely and reliable paracrine Hh function through extracellular diffusion is difficult to envision for two main reasons. First, patterning of folded epithelia, such as *Drosophila* imaginal discs, poses a problem if Hh spreading were to occur out of the plane of the epithelial cell layer through diffusion or flow. The second limitation is that it normally takes much time for diffusing molecules to travel long distances away from the source because the timescale of diffusion increases with the square of the distance [108,109,110] and is inversely correlated with protein size. Both limitations, therefore, also apply to Hh that is permanently associated with soluble Scube2, as described earlier, and to Hh linked to large lipid carriers, as described in the following section (Table 1).

## 7. Proposed Models of Micelle-, Lipophorin- and Membrane-Linked Hh Transport

Although Hh can be solubilized from expressing vertebrate cells in vitro, the majority of Hh in *Drosophila* is found in vesicles in target cells, rather than at the producing cell surface or in the extracellular space [51,111]. This led to several independent models of carrier-dependent extracellular Hh transport. One such model suggests that Hh “pinches off” the plasma membrane and forms micelles to sequester their hydrophobic N- and C-terminal lipids away from the aqueous extracellular environment [40] (Figure 1K). Another model suggests that Hh clusters at the cell surface, through their interaction with glypican HSPGs, recruit and translocate to lipophorin particles (insect apolipoproteins). Lipophorin particles contain various lipidated and GPI-linked proteins, such as Wingless, Hh and glypican; they transport this cargo to target tissues and are endocytosed by the receiving cells [112,113]. More recently, several groups reported Hh transfer by microvesicles or exosomes [114,115,116]. These vesicles are thought to be generated by plasma membrane budding or by fusion of multivesicular bodies with the plasma membrane, which releases intraluminal vesicles into the extracellular space. Evidence for this model also came from RNAi-mediated knockdown of proteins of the endosomal sorting complex required for transport (ESCRT) in Hh-producing cells. Impaired ESCRT function enhanced Hh accumulation at the apical cell surface and decreased long-range signalling in *Drosophila* wing imaginal discs [116]. 

As in the previous models of Scube2-mediated Shh solubilisation, Hh transport on lipophilic carriers generates testable predictions. The first prediction is that soluble Hh should always remain unprocessed and form large multimers that are, because of their lipid content, less dense than water. This prediction derives from, and is a strict consequence of, quantitative Hh cholesteroylation resulting from its unique intein-related maturation, as described earlier. However, biochemical Hh analysis provided ample (yet largely undiscussed) evidence for protein processing, as indicated by different Hh molecular weight fractions on immunoblots [46,81,98,104,114,115,117] and the release of unlipidated monomeric proteins, as judged from gel filtration data [46]. The second important prediction is that general mechanisms of cell-surface protein internalization, sorting and trafficking must have specific adjustments for reliable and robust Hh signalling: It seems unlikely that completely different proteins with specific signalling function are all regulated by the same general mechanisms acting at the same time on the same structures [112,113]. As one such specific adjustment, proposed ESCRT-regulated Hh sorting and internalization for subsequent Hh recycling back to the apical [118,119] or basolateral [120] cell surfaces normally requires cargo recognition via transmembrane and cytoplasmic domains. However, lipid- and GPI-tethered Hh-HSPG clusters lack such domains, which makes the complex “invisible” to the intracellular internalization and sorting machinery. This raises the question of how the established interaction of ESCRT proteins with (ubiquitinylated) cytoplasmic tails is adjusted for Hh internalization and trafficking [114,115,116] and of whether ESCRT proteins interact directly or indirectly with Hh. Other studies showed that the endocytic motor dynamin is not required for Hh transport [54] and that unlabelled Hh visualized by click chemistry in conjunction with proximity ligation assay is not internalized in vitro, as demonstrated by the lack of Hh co-localization with the early endosome marker Rab5a-GFP or the late endosome marker Rab7a-GFP [121]. The final possibility that alternative dynamin-independent endocytosis pathways, such as the clathrin-independent carrier/GPI-AP-enriched early endosomal compartment pathways [122], internalize cell-surface Hh has not been demonstrated so far. We note that one caveat of the observed permanent association of Hh with micelles, lipophorin or trafficking components may be the use of lipidated GFP tags for detection, at least in part, because HhC/ShhC-catalysed GFP cholesteroylation conducted in many of these studies is sufficient to permanently target the protein to cell membranes [123]. 

The basic concept underlying the most prominent model of lipidated Hh transport has been introduced by the Kornberg group, who observed that thin, actin-based filopodia-like extensions carry different signalling proteins over distances of up to several cell diameters in vivo [124,125] (Figure 1L). These specialized extensions, called cytonemes, deliver their cargo to receptors on receiving cells at cellular contacts called “cytoneme synapses” [126]. In larval imaginal wing discs, live cell imaging revealed that cytonemes either emanate from the receiving compartment to take up GFP-labelled Hh from producing cells [127] or from the producing compartment to deliver Hh-GFP to receiving cells [114,128]. In vertebrates and invertebrates, GFP-tagged Hh is transported in the form of large, highly motile cytoneme-associated particles. Yet, because cytonemes represent very thin and highly dynamic structures, it is not clear whether Hh-GFP localizes inside cytonemes or at their surface: Vertebrate ShhN-GFP locates to the exterior of the cytoneme [129], although its fast anterograde and retrograde movements strongly suggest intracellular transport by dynamin motors. Indeed, most recently, intracellular dynamin-dependent transport has been demonstrated for intracellular Hh-GFP vesicles in fly wing disc cytonemes [127].

Despite these ambiguities and despite the multiple steps required for Hh secretion, reinternalization, sorting and transport to and by cytonemes, the cytoneme model fulfils Occam’s razor in two important ways. First, no unnecessary assumptions need to be made about the energy requirements of Hh transport and established lipid biochemistry. Lipids tether Hh to membranes as a prerequisite for cytoneme transport to the receiving cell (Table 1). Second, most cytonemes are impaired in their ability to cross a field of cells made deficient in HSPG expression, revealing a direct functional link between Hh transport and HSPG expression [128]. One important restriction to the model, however, is that non-physiological ShhN-GFP lacking C-cholesterol uses the same route as described for the cholesteroylated protein [129] because cholesteroylated and non-cholesteroylated proteins are both found in large particles within or on cytonemes. In contrast, non-cholesteroylated and untagged Hh is usually monomeric and soluble [40,46]. The cytoneme model alone, therefore, does not explain the functional role of Hh cholesteroylation in Hh biology. Further assumptions must be made to address how, when and where cytoneme length and dynamic regulation is achieved as a necessary prerequisite for specific and robust Hh transport. Finally, an explanation is needed about how lipidated Hhs at cytoneme synapses “switch” between sending and receiving cytonemes, or from sending cytonemes to their receptors on receiving cells [127,130]. 

One solution to the latter problem may come from a direct functional link between Hh lipidation (and subsequent Hh membrane association for cytoneme-mediated transport) and proteolytic Hh release and activation. In this combined mode, Hh processing relays the protein from the cytonemes of producing cells [131] or exosomes [114,132] to the adjacent cytonemes of responding cells [128,129] or to Ptc on the receiving cell surface [133]. Such a proteolytic Hh “relay” over short distances, e.g., in the “cytoneme synapse”, would diminish inherent problems of long-range protein diffusion, as described earlier. Indeed, the logical combination of Hh cytoneme transport to target tissues with a subsequent proteolytic relay was recently indicated by impaired Ptc internalization and function upon binding to irreversibly membrane-linked Hh-CD2 [130]. Hh processing at cytoneme synapses, however, escapes detection by live cell imaging, and the described optimization of cloning protocols for improved in vivo detection of Hh-GFP on membranes via truncation of terminal cholesteroylated peptides [129] may have achieved this goal via impaired Hh processing, resulting in a situation as described for Hh-CD2 [3,130]. Therefore, it is important to determine whether Hh tagging with bulky cholesteroylated GFP can influence Hh PTMs such as HSPG association, multimerization and shedding, and therefore Hh trafficking and function, in vivo.

## 8. Conclusion and Outlook

Occam’s razor admonishes us to choose the simplest from a set of models of a given phenomenon that is in accordance with the evidence. Currently, none of the individual postulated concepts accommodate all the available experimental evidence, but their combination can. We propose a working model in which (Scube2-enhanced) proteolytic processing is preceded by membrane-linked Hh transport to target cells. To support possible links between these models, we need to identify further congruencies between them, ideally by using methods and constructs that expand and complement our current knowledge of Hh release and transport. To this end, results obtained from the visualization of Hh-GFP by advanced light microscopy could be tested biochemically and with untagged Hh proteins, and biochemical studies and concepts of Hh processing await in vivo confirmation. The combined model may also be tested for its ability to relay Hh in a reliable and robust manner over both, short and long ranges in vertebrates and invertebrates. Finally, it may help determine whether model organisms using primary cilia for Hh reception, such as vertebrates, possibly modified cilia such as T-cells, or no cilia such as *Drosophila* require different modes of Hh release and transport.

## Figures and Tables

**Table 1:** Summary of proposed models of Hh release and transport (top) and their compatibility with established biochemical properties of Hh proteins and CUB-domain functions (left). −: incompatible. ?: unknown; +: compatible. Note that combined cytoneme Hh transport to target tissues and proteolytic Hh release at these sites is consistent with, and explains, dual Hh lipidation and membrane association, established Scube2 CUB-domain functions as protease enhancers, the essential yet variable role of N-palmitate for Hh biofunction, differential Hh short- and long-range transport to Ptc-receptors on target cells, and the essential roles of HSPGs in Hh-producing (Shedding) and -receiving (Cytonemes) cells. See text for details.

	Hh Cholesterol Extraction by Scube2	Scube2-Regulated Hh Shedding	Micelle, Lipoprotein, and Exosome Transport	Model of Hh Transport Via Cytonemes
Dual Hh membrane association and multimerization	− Problem: Energy required for Hh release from membrane	+ No energy required, general release mechanism in ECM	− Problem: Energy required for Hh release from membrane	− Problem: required Hh transfer at the cytoneme synapse
Established CUB-domain functions, exergonic Hh release	? CUB binding/extraction of cholesterol endergonic	+ CUBs are established protease regulators	? Not addressed	? Not addressed
Variable role of palmitate and functionality of other hydrophobic Hh modifications	? Not addressed	+ Processing of lipidated termini activates Hh, lipids function indirectly as membrane tethers	? Not addressed	? Not addressed
Short-range and long-range Hh transport	− Problem: Subsequent diffusion-based transport is not sufficient	− Problem: Subsequent diffusion-based transport is not sufficient	− Problem: Subsequent diffusion-based transport is not sufficient	+ Regulated transport, no long-range diffusion required
HSPGs in Hh release and transport	? Not addressed	+ Recruit Scube2 and generate release hubs (in producing compartment)	+ Permissive factors for Hh transfer and transport	+ Permissive factors for cytoneme extension (in receiving compartment)

## Abbreviations

Hh	Hedgehog
Shh	Sonic hedgehog
HhC	C-terminal autoprocessing/cholesterol transferase domain
Hh/Shh	fully bioactive, dual-lipidated proteins
HhN/ShhN	non-cholesteroylated artificial forms of invertebrate Hh/vertebrate Shh due to deletion of HhC/ShhC ShhC25S represent C-cholesteroylated, N-terminally unpalmitoylated protein
PTM	posttranslational protein modification
ECM	extracellular matrix
Ptc	Patched
GPI	glycosylphosphatidyl-inositol
ER	endoplasmic reticulum
Disp	Dispatched
Hhat	Hedgehog acyltransferase, also called Ski (Skinny hedgehog)
EGF	epidermal growth factor
Scube	Signal sequence, cubulin domain, EGF-like growth factor domain
HS	heparan sulfate
HSPG	HS proteoglycan
PCPE	procollagen C-proteinase enhancer
MO	morpholino
GFP	green fluorescent protein
ESCRT	endosomal sorting complex required for transport
